# Ubiquitin-Proteasome System in the Different Stages of Dominantly Inherited Alzheimer’s Disease

**DOI:** 10.21203/rs.3.rs-4202125/v1

**Published:** 2024-07-23

**Authors:** Eric McDade, Haiyan Liu, Quoc Bui, Jason Hassenstab, Brian Gordon, Tammie Benzinger, Yuanyuan Shen, Jigyasha Timsina, Lihua Wang, Yun Ju Sung, Celeste Karch, Alan Renton, Alisha Daniels, John Morris, Chengjie Xiong, Laura Ibanez, Richard Perrin, Jorge J Llibre-Guerra, Gregory Day, Charlene Supnet-Bell, Xiong Xu, Sarah Berman, Jasmeer Chhatwal, Takeshi Ikeuchi, Kensaku Kasuga, Yoshiki Niimi, Edward Huey, Peter Schofield, William Brooks, Natalie Ryan, Mathias Jucker, Christoph Laske, Johannes Levin, Jonathan Vöglein, Jee Hoon Roh, Francisco Lopera, Randall Bateman, Carlos Cruchaga

**Affiliations:** Washington University in St. Louis; Washington University in St. Louis; Washington University in St. Louis; Washington University Medical School; Washington University in St. Louis; Nash Family Department of Neuroscience and Ronald Loeb Center for Alzheimer’s Disease, Icahn School of Medicine at Mount Sinai, New York, NY, USA: Departments of Neurology and Genetics and Ge; Washington University School of Medicine; Washington University in St. Louis; Mayo Clinic in Florida; Massachusetts General Hospital, Brigham and Women’s Hospital, Harvard Medical School; Niigata University, Brain Research Institute; Department of Molecular Genetics, Brain Research Institute, Niigata University; Neuroscience Research Australia; University of Tübingen; Eberhard-Karls University Tübingen; Ludwig-Maximilians-Universität; Korea University College of Medicine; Department of Neurology, Washington University School of Medicine; Washington University

## Abstract

This study explored the role of the ubiquitin-proteasome system (UPS) in dominantly inherited Alzheimer’s disease (DIAD) by examining changes in cerebrospinal fluid (CSF) levels of UPS proteins along with disease progression, AD imaging biomarkers (PiB PET, tau PET), neurodegeneration imaging measures (MRI, FDG PET), and Clinical Dementia Rating^®^ (CDR^®^). Using the SOMAscan assay, we detected subtle increases in specific ubiquitin enzymes associated with proteostasis in mutation carriers (MCs) up to two decades before the estimated symptom onset. This was followed by more pronounced elevations of UPS-activating enzymes, including E2 and E3 proteins, and ubiquitin-related modifiers. Our findings also demonstrated consistent correlations between UPS proteins and CSF biomarkers such as Aβ42/40 ratio, total tau, various phosphorylated tau species to total tau ratios (ptau181/T181, ptauT205/T205, ptauS202/S202, ptauT217/T217), and MTBR-tau243, alongside Neurofilament light chain (NfL) and the CDR^®^. Notably, a positive association was observed with imaging markers (PiB PET, tau PET) and a negative correlation with markers of neurodegeneration (FDG PET, MRI), highlighting a significant link between UPS dysregulation and neurodegenerative processes. The correlations suggest that the increase in multiple UPS proteins with rising tau levels and tau-tangle associated markers, indicating a potential role for the UPS in relation to misfolded tau/neurofibrillary tangles (NFTs) and symptom onset. These findings indicate that elevated CSF UPS proteins in DIAD MCs could serve as early indicators of disease progression and suggest a link between UPS dysregulation and amyloid plaque, tau tangles formation, implicating the UPS as a potential therapeutic target in AD pathogenesis.

## 1. Introduction

Alzheimer’s disease (AD) is a multifactorial disorder influenced by a variety of genetic and environmental factors.^1^ The disease is characterized by the accumulation of misfolded, insoluble protein aggregates, composed primarily of amyloid-β (Aβ) peptide (plaques) and phosphorylated tau protein (forming neurofibrillary tangles (NFT)) in the brain ^2,3^, which leads to the insidious onset and gradual disruption of cognitive and behavioral functions^,3,4,5^.

Recent studies highlight the role of faulty proteostasis in the progression of neurodegenerative diseases ^6, 7,8^. Proteostasis encompasses cellular mechanisms that regulate protein synthesis, folding, post-translational modification, and degradation, mechanisms that are disrupted in conditions like AD ^6, 9,10,11^. The ubiquitin-proteasome system (UPS) and the autophagy lysosomal pathway work in tandem to preserve proteostasis in cells by preventing the accumulation of non-functional and misfolded proteins ^12,13,14^. UPS degrades substrates that are potentially toxic by breaking them down into small peptides to replenish intracellular amino acid pools^15^. In humans, the UPS consists of two activating enzymes (E1s), approximately 40 conjugating enzymes (E2s), more than 600 ligase enzymes (E3s), and approximately 100 deubiquitinases (DUBs) ^16, 17, 18^. Proteostasis defects can lead to neuronal stress, synapse loss, and memory deficits such that impaired proteostasis is considered a main contributor to AD pathogenesis ^8^.

The association between proteasomal dysfunction and AD was first established through histopathological examinations, which highlighted the accumulation of ubiquitin in AD-associated plaques and tangles ^19,20,21^. Subsequent Genome-Wide Association Studies (GWAS) and proteomic studies have corroborated this link by identifying key roles for the proteasomal pathway in patients with *symptomatic* AD and transgenic AD models ^19, 22,23,24, 25, 26^. These advanced methodologies uncovered significant changes at the proteome level during AD progression, particularly highlighting the dysregulation of the UPS ^26^. This dysregulation is characterized by changes in the levels of certain ubiquitin-activating and ubiquitin-conjugating enzymes, coupled with the accumulation of a mutant form of ubiquitin known as UBB + 1, due to genetic alterations. These alterations lead to the inhibition of proteasome activity ^27,28^. Additionally, the dysregulation of ubiquitin-mediated pathways is associated with alterations in learning and memory ability, Aβ plaque formation, hyperphosphorylation of tau protein, as well as synaptic plasticity and immune function changes in AD mouse models^19,20, 21^. The potential therapeutic implications of these findings are underscored by the promising effects of small molecules targeting the proteasomal pathway in animal and cellular models of AD ^29^. To date, most studies of the UPS have been undertaken using animal or cellular models of AD or in brain tissue of symptomatic AD cases. Given the recent evolution of methods for studying AD pathology biomarkers in humans, there is now the opportunity to evaluate the role of the UPS system in the presymptomatic and symptomatic stages of AD.

Studies in Dominantly Inherited Alzheimer’s Disease (DIAD) allow the examination of disease-related proteins from the presymptomatic stage to moderately symptomatic stages of AD over three decades of disease progression. Here we analyzed cerebrospinal fluid (CSF) proteomic data from DIAD individuals. Leveraging the high-throughput capabilities of the SOMAscan proteomics platform and data from the Dominantly Inherited Alzheimer Network (DIAN), we explored the changes in expression, stability, and modifications of UPS proteins throughout the disease course. Considering existing evidence that abnormal accumulation of Aβ and tau proteins in the brain in AD begins well before the onset of neurological symptoms, up to 20 years prior, we investigated the early accumulation of both Aβ and tau aggregated protein species in relation to UPS dysregulation in DIAD ^28, 30, 31, 32,33^. We aimed to explore if dysregulation of UPS proteins impacts the progression of DIAD by assessing the associations with Aβ, and tau pathologies, neuronal loss, and neuroinflammation (all measured using existing established CSF and neuroimaging biomarkers) and clinical symptoms. Our findings could provide important insights into AD initiation and progression and potentially reveal novel biomarkers of disease progression and new therapeutic targets.

## 2. Methods

### 2.1 Participants

The DIAN observational study (DIAN Obs) recruited participants from families that carry an autosomal-dominant Alzheimer’s disease mutation in one of three genes - *APP, PSEN1*, or *PSEN2*. DIAN Obs is a longitudinal, observational study in which participants undergo comprehensive assessments including clinical and neuropsychological testing, brain imaging, and collection of biofluids such as CSF and blood^34–37^. This analysis incorporated cross-sectional clinical data and CSF measures in 289 mutation carriers (MCs) and 172 mutation non-carrier participant controls (NC) from data freeze-15, each with at least one CSF measure^38^. Mutation status was determined using PCR-based amplification of the relevant exon(s) followed by Sanger sequencing ^32^.

All procedures were approved by the institutional review board at Washington University in St. Louis. Written informed consent was obtained from participants or their caregivers, adhering to the guidelines of their respective local institutional review boards. To ensure participant confidentiality and due to the limited number of individuals at the extreme ends of the timeline, we have not displayed individual participant data for the period before − 30 years and after 10 years of estimated symptom onset.

### 2.2 CSF Sample Collection and protein measurements by SOMAscan

Cerebrospinal fluid (CSF) samples were collected after an overnight fast and preserved at −80°C for subsequent protein level measurements using the Slow Off-rate Aptamer (SOMAmer)-based capture array, SOMAscan^39^. Protein measurements reported in relative fluorescence units (RFU) underwent hybridization, median, and iterative adaptive normalization by maximum likelihood (ANML) procedures until convergence. Ensuring data integrity, we performed an in-house quality control, excluding aptamers shared by approximately 70% of participant sample outliers^39^.

All proteins of interest were analyzed using the SOMAscan assay (v4.1) from SomaLogic^39^. To identify UPS proteins within our SOMAscan dataset, the UniProt representational state transfer (REST) application programming interface (API) was employed to cross-reference our dataset with the UPS category in UniProt’s controlled vocabulary. Further refinement was achieved using the fetching annotations from UniProt and Reactome databases. Discrepancies were manually verified for accuracy. We identified 174 UPS proteins from a SOMAscan pool of approximately 6600 proteins for further analysis.

### 2.3 Immunoassay

The levels of Aβ42, Aβ40, and total tau (t-tau) in CSF were measured using the Lumipulse platform (Fujirebio, Tokyo, Japan) through immunoassay techniques^40^; Phosphorylated to unphosphorylated ratio of tau at threonine 181, 205, and 217, serine 202 (pT181/T181, pT205/T205, pT217/T217 and pS202/S202) in CSF were measured by mass spectrometry ^41,42^. CSF Soluble triggering receptor expressed on myeloid cells 2 (TREM2) immunoassay was performed as described previously ^43,40^. Neurofilament light chain (NfL) levels were measured in CSF and serum using enzyme-linked immunosorbent assay(ELISA )^44,45,46^.

### 2.4 Clinical Assessment and DIAN Estimated Year from Symptom Onset

The Clinical Dementia Rating–Sum of Boxes (CDR^®^-SB) assessment scale was used to assess the stage of dementia in a blinded manner by clinical evaluators (The scale ranges from 0 to 18, with higher scores denoting more significant impairment) ^47^. The participant’s estimated years from symptom onset (EYO) were calculated at each visit based on their age and expected age of symptom onset specific to their mutation. If this information was unavailable, the EYO was calculated at the age at which parental cognitive decline began, as determined through a semi-structured interview and historical data ^48^.

### 2.5 Imaging

Imaging included Magnetic Resonance Imaging (MRI) and positron emission tomography (PET) imaging for volumetric analyses as well as evaluations of amyloid- β (using ^11^C-Pittsburgh Compound B (PiB) PET) and glucose metabolism (using 18F-FDG-PET) as detailed previously ^36^. Using FreeSurfer 5.3, we defined cortical and subcortical regions of interest (ROIs). Both PET modalities were partially volume-corrected via a regional spread function technique ^49,50,44^. Our study concentrated on the precuneus region for its early and consistent involvement by AD pathology in DIAD ^32,50,51^. Tau PET imaging utilized 18F-AV-1451 (flortaucipir), with data from the 80–100 min window converted to Standardized Uptake Value Ratios (SUVRs). To address differences in scanner spatial resolutions, scanner-specific spatial filters were applied, standardizing to a common resolution of 8 mm Region of Interest (ROI) PET data were also converted to SUVRs using the cerebellar grey matter as a reference. Partial volume correction was implemented using a regional spread function for each region, forming a geometric transfer matrix ^52,53,54,55^.

### 2.6 Statistical analysis

In our study, cross-sectional analyses were conducted to examine the descriptive characteristics and baseline biomarker values across distinct clinical groups. These analyses employed chi-square (χ2) tests to assess differences in categorical variables and Analysis of Variance (ANOVA). This approach facilitated a detailed investigation of baseline biomarker discrepancies among the groups. Furthermore, we categorized mutation carrier participants into two distinct cohorts: asymptomatic carriers (those with a baseline CDR^®^ score of 0) and symptomatic carriers (those with a baseline CDR^®^ score greater than 0).

The cross-sectional relationship of different levels of UPS protein between the two mutation groups along the DIAN EYO was evaluated using a linear mixed-effects (LME) model. This model included fixed effects of the mutation group, EYO, and the interaction between mutation groups and EYO, along with random intercepts at the family level. Subsequently, a comparison of the estimated UPS levels between the two groups at each value of DIAN EYO, ranging from − 30 to + 10, was conducted. The EYO point at which the differences became statistically significant was determined by contrasting with specific EYO points. These estimators were then plotted against baseline EYO using local regression (LOESS).

Partial correlation analysis adjusting for age was conducted to assess the correlation between UPS proteins and each biomarker in each mutation group. Then estimated correlation coefficients were compared using Fisher’s Z transformation. Because of the large number of pairwise correlations to be compared, we controlled the False Discovery Rate (FDR) at 5% level ^56^. Additionally, Analysis of Covariance (ANCOVA) for continuous variables were used to assess the differences between the NC and MC groups, taking age, sex, and *APOE* ε4 status into account as covariates, while also maintaining the FDR control at the 5% level.

Statistical analyses were performed using SAS version 9.4 (SAS Institute) and plots were created with RStudio (version 4.3.1). P values were obtained through two-tailed tests, adopting a significance threshold of p < 0.05 to determine statistical significance.

## 3. Results

### 3.1 Participants Demographics

The cross-sectional cohort study included 179 asymptomatic mutation carriers (MCs) with an average age of 35.6 years (SD = 8.6) and an estimated years to onset (EYO) of −13.4 years (SD = 8.6), 104 symptomatic mutation carriers (MCs) with an average age of 47.4 years (SD = 9.0) and an EYO of 4.1 years (SD = 2.9), and 172 asymptomatic mutation non-carriers (NCs) with an average age of 39.2 years (SD = 11.4). The NCs were, on average, −9.0 years (SD = 12.2) younger relative to the EYO of their MC siblings. Comprehensive demographic details and baseline characteristics of the participants, as well as fluid and imaging biomarkers, are summarized in Table 1.

### 3.2 UPS Proteins Changes in CSF

**Figure 1:**
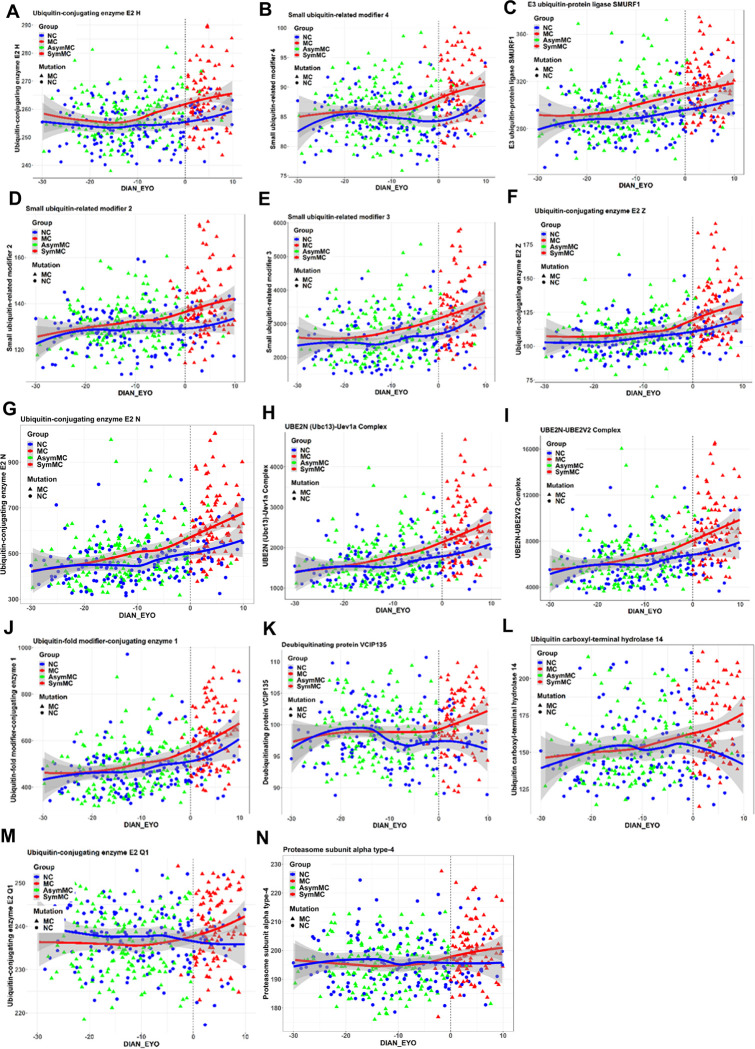


Our LME model analysis identified a significant increase in CSF levels of 14 proteins when comparing MC to NC across EYO. These proteins encompassed six E2 enzymes (ubiquitin-conjugating enzymes), one E3 enzyme (ubiquitin ligase), four ubiquitin modifiers, two deubiquitinases, and one proteasome component, all showing statistical significance with FDR p-values less than 0.05 (Fig. 1). Notably, in MCs, the cross-sectional levels of certain proteins within the ubiquitin pathway began to elevate nearly two decades before the EYO. Specifically, between 15 and 20 years prior to the EYO, significant increases were observed in proteins such as ubiquitin-conjugating enzyme E2 H (UBE2H), the E3 ubiquitin ligase SMURF1 (SMURF1), and the small ubiquitin-related modifiers 2, 3, and 4 (SUMO2, SUMO3, and SUMO4).

Between 10 and 15 years prior to symptom onset, multiple proteins within the UPS, particularly E2 ubiquitin-conjugating enzymes, began to show significant increases in MC compared to NC. These increases included ubiquitin-conjugating enzyme E2 Z (UBE2Z), ubiquitin-conjugating enzyme E2 N (UBE2N), the UBE2N/Ubiquitin-conjugating enzyme E2 variant 1A (Uev1a) complex, the UBE2N/Ubiquitin-conjugating enzyme E2 variant 2 (UBE2V2) complex, ubiquitin-fold modifier-conjugating enzyme 1 (UFC1), and the deubiquitinating protein VCIP135. Furthermore, within the 10 to 0-year period leading up to symptom onset, alterations were observed in ubiquitin carboxyl-terminal hydrolase 14 (USP-14), ubiquitin-conjugating enzyme E2 Q1 (UBE2Q1), and the proteasome subunit alpha type-4 (PSMA4). The specific years showing significant differences are depicted in Fig. 1. No modifying effects were observed based on sex, education level, or *APOE* ε4 status. Of note, nearly all these UPS proteins demonstrated the greatest difference between symptomatic MCs and NCs as symptoms progressed, suggesting a continuing rise with disease progression.

### 3.3 Partial Spearman’s Rank Correlation Analysis of UPS proteins and AD biomarkers

#### 3.3.1 Correlation analysis with amyloid related biomarkers and amyloid PET

##### 3.3.1.1 Correlation with Amyloid PET

**Figure 2:**
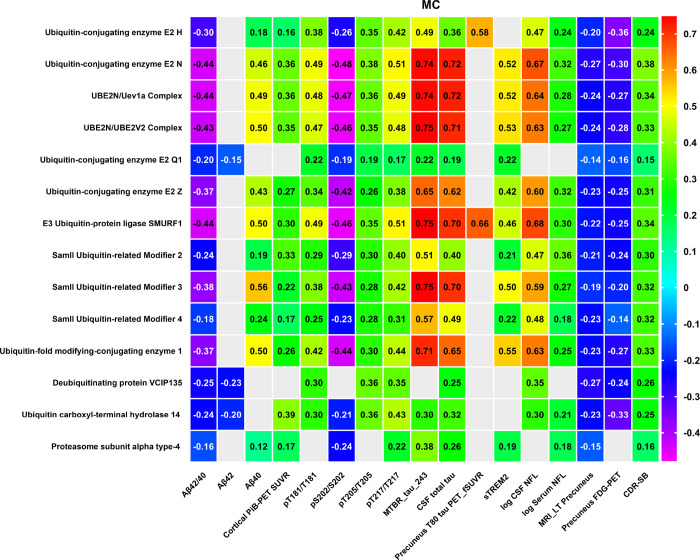


After adjusting for age and sex, our analysis indicated that most the above 14 UPS proteins (result 3.2) demonstrated mild to moderate correlations with cortical amyloid PET (PiB PET) SUVR in the MC group, in contrast to the NC group. The correlation coefficients varied from 0.16 to 0.39. Specifically, proteins such as UBE2N, UBE2N/Uev1a, UBE2N/UBE2V2, SMO2, E3 ubiquitin ligase SMURF1, and USP-14 showed significant differences between the MC and NC groups (FDR p < 0.05). For more information, please see Fig. 2 and Table 3.

#### 3.3.2 Correlation with amyloid related biomarkers

The correlations of the 14 UPS proteins were primarily with soluble CSF Aβ. Notably, these correlations were significantly inversely related to the Aβ42/40 ratio in the MC group, with r values ranging from −0.16 to −0.44. This pattern is largely attributed to a positive association with Aβ40. While NCs also demonstrated several associations with soluble Aβ, proteins such as UBE2N, UBE2N/Uev1a, UBE2N/UBE2V2, and UFC1 positively correlated with Aβ42. In contrast, the associations with Aβ40 were more pronounced in NCs compared to MCs. For additional information, please refer to Fig. 2 and Table 3.

#### 3.3.3 Correlation with tau related biomarkers

##### 3.3.3.1 Correlation with tau PET

We also evaluated the associations between those 14 UPS proteins and flortaucipir uptake in the precuneus for both MC and NC, utilizing Spearman correlation models adjusted for age and sex. Proteins including UBE2H and E3 ubiquitin-protein ligase SMURF1 demonstrated moderate to strong associations with an increasing tau PET signal in the precuneus, with r values ranging from 0.58 to 0.66 in the MC group (p < 0.05, FDR 5%). No significant association was observed in the NC group.

##### 3.3.2.2 Correlation with soluble CSF tau-related biomarkers

We identified significant correlations between CSF total tau in both the MC and NC groups, with each group showing a substantial correlation. These proteins including UBE2N, UBE2N (Ubc13)/Uev1a Complex, UBE2N/UBE2V2 Complex, UBE2H, UBE2Q1, UBE2Z, E3 ubiquitin-protein ligase SMURF1, SUMO2, SUMO3, SUMO4, UFC1, USP14, Deubiquitinating protein VCIP-135 andPSMA4, exhibited stronger correlation coefficients in MC group, ranging from approximately 0.25 to 0.72 (p < 0.05, FDR 5%)). (See Fig. 2 and Table 3 for details.

In the MC group, all of the aforementioned 14 UPS proteins, with the exception of PSMA4, exhibited a positive correlation with pTau181/T181, with r values ranging from 0.22 to 0.49, and with pTau205/T205, where r values ranged from 0.19 to 0.38. Furthermore, these 14 UPS proteins also showed a positive correlation with pTau217/T217, with r values spanning from 0.17 to 0.51. Conversely, 13 out of the 14 UPS proteins, excluding the deubiquitinating protein VCIP-135, demonstrated a negative correlation with pS202/S202, with correlation coefficients ranging from approximately − 0.21 to −0.48.

The most significant correlations were observed with 13 of these 14 proteins, excluding the deubiquitinating protein VCIP-135 and MTBR-tau243, in both MC and NC groups. Their r values varied from approximately 0.3 to 0.75 in the MC group. Although statistically significant correlations were identified in NCs, those in MCs with 3.3–10 times greater based on the model estimated correlations, the β coefficients ranging from 0.000098 to 0.02581 (MC vs NC). Notably, the deubiquitinating protein VCIP-135 was negatively associated with MTBR-tau243, with an r value of −0.41 in the NC group. Table 3 outlines the absolute differences in beta coefficients between MC and NC groups. For further details, refer to Fig. 2 (MC) and Table 3 (MC vs NC).

#### 3.3.3 Correlation analysis with neurodegeneration and clinical state

To investigate the relationship between CSF UPS protein levels and imaging markers of neurodegeneration, as well as clinical stages, we conducted correlation analyses with various imaging parameters. These included FDG Composite and MRI based precuneus cortical thickness, alongside the CDR^®^-SB. Acknowledging the established correlation between age, sex, AD disease stage, and the age-associated increase in numerous proteostasis peptides, we adjusted the correlations for both age and sex ^48,57,58^. Our findings reveal that all 14 UPS proteins exhibited a significant, though mild to moderate, positive correlation with CDR^®^-SB sin the MC (see Fig. 2). Moreover, all UPS proteins, except PSMA4, demonstrated mild to moderate negative correlations with FDG PET in the precuneus region for the MC group, with r values ranging from − 0.14 to −0.36. Additionally, all 14 UPS proteins displayed a mild negative correlation with MRI findings in the precuneus region (left), with r values ranging from − 0.14 to −0.27.

#### 3.3.4 Correlation with CSF and Serum Neurofilament Light Chain (NfL)

We identified a significant positive correlation between the logarithmic values (log) of CSF and serum NfL. Notably, within the MC impairment group, CSF NfL demonstrated moderate to high positive correlations with 12 of the 14 UPS proteins discussed in section 3.2, excluding UBE2Q1 and PSMA4. The correlation coefficients (r) ranged from 0.3 to 0.68. In the NC group, UBE2N, UBE2N/Uev1a, UBE2N/UBE2V2, UBE2Z, and UFC1 also showed positive correlations with CSF NfL, albeit the associations were more marked in the MC group compared to the NC group. Furthermore, 12 out of these 14 proteins, with the exception of UBE2Q1 and the deubiquitinating protein VCIP-135, exhibited mild to moderate positive correlations with serum NfL in the MC group, with correlation coefficients ranging approximately from 0.18 to 0.36.

##### 3.3.2.4 Correlation with Soluble TREM2

We observed a significant positive correlation between the normalized levels of CSF sTREM2 (normalized using an internal standard, termed relative sTREM2) and a selection of 14 UPS proteins in both MC and NC groups. In the MC group, all proteins except UBE2H, USP-14, and VCIP-135 showed positive correlations with sTREM2, with r-values ranging approximately from 0.21 to 0.55. In the NC group, proteins such as UBE2N, the UBE2N/Uev1a Complex, the UBE2N/UBE2V2 Complex, UBE2Z, E3 ubiquitin-protein ligase SMURF1, SUMO3, and UFC1 also displayed positive correlations with sTREM2, with r values ranging from 0.43 to 0.56, which reached statistical significance (p < 0.05, FDR 5%). However, Fig. 1 illustrates that NCs maintain very normal levels for nearly all these 14 proteins, suggesting that the observed correlations might be driven by much smaller variance rather than a greater range. Furthermore, the model-estimated correlations analysis in Table 3 indicated that the β coefficients showed no significant difference between MC and NC for all 14 UPS proteins. This observation suggests that the associations may not necessarily reflect biological phenomena but could instead be attributed to characteristics of the assay.

## Discussion

In our study, we observed that levels of 14 UPS proteins were elevated in the MC group compared to the NC group across different stages of DIAD. Notably, these differences were especially significant around the time of predicted clinical symptom onset and persisted beyond this point (Fig. 1). The presence of specific DIAD mutations in either the *PSEN1, PSEN2*, or *APP* genes. did not significantly affect the extent of the increase in UPS protein levels.

Furthermore, our findings reveal consistently stronger associations with MTBR-tau243, total tau, tau PET, and CSF NfL. Additionally, the correlations between rising levels of UPS-related proteins and markers of neurodegeneration, as evidenced by PiB PET, FDG PET, and MRI, underscore the significant link between UPS dysregulation and neurodegenerative processes. Our results also indicate a consistent correlation between UPS protein levels and several CSF biomarkers, including phosphorylated tau (ptauT181/T181, ptauS202/S202, ptauT205/T205, ptauT217/T217), the Aβ 42/40 ratio, sTREM2, serum NfL, and CDR^®^-SB. These correlations suggest that amyloid aggregation is accompanied by an increase in these UPS proteins, but the increase is more pronounced when tau levels rise, suggesting the potential involvement of the UPS in the pathogenesis of AD, particularly in relation to misfolded tau/ NFT and critical disease progression points leading to symptom onset.

### UPS, Autophagy and AD

**Figure 3:**
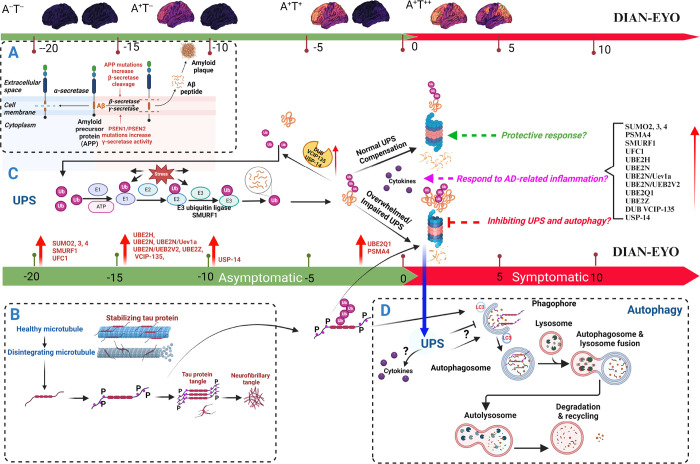


The UPS and autophagy represent crucial protein degradation pathways in eukaryotes, targeting distinct substrate types. The UPS primarily processes short-lived, misfolded soluble proteins, while autophagy addresses longer-lived proteins, insoluble aggregates, and organelles. Both pathways utilize ubiquitin for target recognition, underscoring their significance in cellular health and proteostasis maintenance^59,60,61,62,66^. Notably, the E3 ubiquitin-protein ligase SMURF1 plays a crucial role in autophagy regulation by activating PPP3/calcineurin and TFEB, highlighting the lysosome’s significant role in cell signaling. SMURF1 impacts lysosomal biogenesis and, together with PPP3/calcineurin, governs the autolysosome pathway, indirectly aiding in autophagosome maturation through TFEB regulation^63^. In our study, we noted an upregulation of SMURF1 in DIAD, suggesting it might act as a protective mechanism to enhance protein quality control or could directly contribute to AD pathogenesis through autophagy regulation. The pathophysiological significance of SMURF1 in DIAD, along with its potential interactions with autophagy in AD whether direct or indirect warrants further exploration to elucidate their impact on disease progression. The involvement of the UPS in DIAD and the possible mechanisms are depicted in Fig. 3.

### Enzymes E2, E3 Enzymes and AD and Other Neurodegenerative Disease

Alterations in the Ube2 subfamily genes, notably UBE2N, play a significant role in AD and other neurodegenerative disorders^68, 69^. Changes in the expression and methylation of *UBE2N* and its complexes suggest their involvement in AD pathology, such as protein aggregation and genomic regulation^64, 65^.. Recent research using Gene Expression Omnibus (GEO) data identified UBE2N as an immune-related biomarker for AD, linked to T cell and B cell functions and synaptic signaling^66^. Suppressing UBE2N has been shown to alleviate AD pathology by enhancing amyloid-β clearance in mouse models, marking it as a potential therapeutic target^67^. Moreover, heterodimers like UBE2V1-a, involved in atypical polyubiquitination, impact inflammation and proteasomal degradation^67,65,68^. Other UBE2 enzymes, such as UBE2I, UBE2Q1, UBE2E1, and UBE2Z, display varied regulatory patterns in neurodegenerative diseases like frontotemporal dementia, suggesting the Ube2 family’s extensive influence on neurodegeneration, inflammation, and cellular stress responses^69^.

Our study highlights significant changes in E3 ubiquitin ligases, especially SMURF1, which is associated with aggresome formation in AD, a mechanism to prevent the toxic spread of misfolded proteins^70^. SMURF1’s localization in Hirano bodies suggests its involvement in neurodegeneration^71^. Elevated levels of UBE2 family proteins and SMURF1, observable years before AD symptoms appear, suggest their early role in AD pathogenesis and potential as biomarkers. Their increased levels post-symptom onset, correlating with neurodegeneration markers like NfL and aggregated-tau, underscore their importance in neurofibrillary tangle development and neurodegeneration^72,70,73,74^. This underlines the need for further explore the Ube2 family and SMURF1’s roles in AD progression and their therapeutic possibilities.

### Ubiquitin Modifiers and AD

Ubiquitin-fold modifier conjugating enzyme 1 (UFC1) is significantly associated with AD, playing a crucial role in protein folding, secretion, and endoplasmic reticulum (ER) stress. Elevated UFC1 levels in the CSF of individuals with mild cognitive impairment in sporadic AD suggest its involvement in AD’s pathogenesis ^75,76^. Our study supports this finding, showing a strong positive correlation between UFC1 and CSF NfL and a moderate correlation with total tau, marker of later stages of disease^77^.

Additionally, our study enhances understanding of post-translational modifications in AD through the role of SUMOs and SUMOylation ^74, 78,79, 80^. We observed increased levels of SUMO2, SUMO3, and SUMO4 in individuals with DIAD mutations, implicating SUMOylation in AD pathophysiology. This modification of AD-related proteins, such as APP, affects amyloid-β aggregation, with certain mutations potentially exacerbating the disease. Moreover, SUMOylation’s involvement in tau phosphorylation suggests its impact on tau stability and degradation, contributing to AD’s characteristic neurofibrillary tangles and neuronal loss ^74, 79, 80^. The upregulation of SUMO proteins in DIAD MCs before symptom onset suggests SUMOylation’s role in early AD pathology, warranting further exploration to understand its mechanisms and aging-related effects. Our findings suggest that modulating SUMOylation processes may offer early detection biomarkers and new therapeutic targets in AD.

### Deubiquitinase and AD

In AD, alterations in DUBs underscore their critical role in maintaining ubiquitination balance and their potential involvement in disease progression^74,79, 81, 82,83^. Dysregulated DUBs are implicated in neuropathy and neurodegeneration; for instance, USP-14 is linked to neuromuscular dysfunctions, while UCHL-1 levels correlate with key AD biomarkers and cognitive scores^79, 81, 84^. Our study notably identified elevated levels of deubiquitinating protein VCIP-135 and USP-14, which are involved in cellular homeostasis and protein processing, within the group. This elevation might suggest a compensatory mechanism in response to the misfolded proteins characteristic of AD or a role in the proteasomal degradation process^85,86, 87,88^. However, our findings did not reveal a significant role for UCHL-1 in AD progression, indicating that its involvement may vary across different stages of the disease.

### Proteasome and AD

The UPS, with the proteasome as its essential component, is crucial for degrading ubiquitinated proteins. The proteasome is a barrel-shaped 20S complex composed of four types of subunits (α, β, γ, δ), with the β-subunits having peptide-cleaving capabilities^82^. Post-mortem examinations of AD brains have shown reductions in caspase-like and chymotrypsin-like proteasome activities^89^. Protein oxidation, often discussed as a factor in AD progression, remains debated as either a cause or consequence of the pathology. Protein oxidation and excessive phosphorylation could impede the proteasome’s key roles in intracellular protein quality control and the processing of Aβ and tau, potentially influencing AD pathology^90^. The proteasome’s role in AD remains critically underexplored. Our study highlights the differential expression of *PSMA4* between MC and NC groups around symptom onset, with this difference increasing as the disease progresses. This underlines the critical need for more research into the interplay between genetic variations, proteasome function, and neurodegenerative disorders.

### Strengths and Limitations

This study represents a foundational investigation into the UPS in AD, leveraging multi-modal data from a detailed cohort of DIAD participants. Despite its strengths, including the use of sensitive proteomics and corroborative imaging, the study faces limitations. It focuses on DIAD, whose genetic predictability differs from the more common sporadic AD, potentially limiting the generalizability of our findings. The cross-sectional design restricts our ability to infer causality or the sequence of UPS changes relative to disease progression, pointing to the need for longitudinal studies. Additionally, our proteomic analysis, limited to proteins detectable by the SOMAscan assay, might not capture all relevant UPS alterations, nor does it clarify the implications of extracellular versus intracellular protein levels. Future research should expand the range of UPS proteins analyzed and compare DIAD to sporadic AD to enhance our understanding of the UPS in AD pathophysiology.

### Conclusion

our study underscores UPS dysregulation in DIAD, particularly highlighting the upregulation of the UBE2 family, E3 ligase SMURF1, ubiquitin modifiers like SUMO2, 3, 4, UFC1, deubiquitinase USP-14, VCIP-135, and proteasome component PSMA4. This upregulation, emerging 10 to 15 years before symptom onset and coinciding with increases in Aβ, tau, phospho-tau, and tau PET findings, offers insights into AD mechanisms. It may reflect a protective response or contribute to AD pathogenesis, possibly in reaction to AD-related inflammation^51, 91, 92,93^. These findings necessitate further research to explore these proteins’ roles in misfolded protein aggregation and their impact on other degradation systems like autophagy^94^. The link between UPS alterations and tau pathology suggests a connection to disease progression and late-stage biomarkers. Our study, highlighting correlations with Aβ, tau load, brain volume, and metabolic changes, opens new research directions for therapeutic strategies targeting UPS to reduce protein aggregation and inflammation in AD. This emphasizes the importance of further exploration into the UPS’s involvement in DIAD pathogenesis.

## Data Availability

Due to the rarity of dominantly inherited Alzheimer’s disease, individual-level data from DIAN cannot be shared publicly, as it would compromise participant anonymity. This limitation has been validated by the Institutional Review Board (IRB) and confirmed with the NIH. Nevertheless, this data remains accessible for qualified researchers upon request. Requests can be submitted through the following link: DIAN Biospecimen Request Form.

## DIAN Consortium List:

Randall J. Bateman, James M. Noble, Gregory S. Day, Neill R. Graff-Radford, Jonathan Vöglein, Ricardo Allegri, Patricio Chrem Mendez, Ezequiel Surace, Sarah B. Berman, Snezana Ikonomovic, Neelesh Nadkarni, Francisco Lopera, Laura Ramirez, David Aguillon, Yudy Leon, Claudia Ramos, Diana Alzate, Ana Baena, Natalia Londono, Sonia Moreno, Mathias Jucker, Christoph Laske, Elke Kuder-Buletta, Susanne Graber-Sultan, Oliver Preische, Anna Hofmann, Takeshi Ikeuchi, Kensaku Kasuga, Yoshiki Niimi, Kenji Ishii, Michio Senda, Raquel Sanchez-Valle, Pedro Rosa-Neto, Nick Fox, Dave Cash, Jae-Hong Lee, Jee Hoon Roh, Meghan Riddle, William Menard, Courtney Bodge, Mustafa Surti, Leonel Tadao Takada, Martin Farlow, Jasmeer P. Chhatwal, V. J. Sanchez-Gonzalez, Maribel Orozco-Barajas, Alison Goate, Alan Renton, Bianca Esposito, Celeste M. Karch, Jacob Marsh, Carlos Cruchaga, Victoria Fernandez, Brian A. Gordon, Anne M. Fagan, Gina Jerome, Elizabeth Herries, Jorge Llibre-Guerra, Allan I. Levey, Erik C. B. Johnson, Nicholas T. Seyfried, Peter R. Schofield, William Brooks, Jacob Bechara, Eric McDade, Jason Hassenstab, Richard J. Perrin, Erin Franklin, Tammie L. S. Benzinger, Allison Chen, Charles Chen, Shaney Flores, Nelly Friedrichsen, Nancy Hantler, Russ Hornbeck, Steve Jarman, Sarah Keefe, Deborah Koudelis, Parinaz Massoumzadeh, Austin McCullough, Nicole McKay, Joyce Nicklaus, Christine Pulizos, Qing Wang, Sheetal Mishall, Edita Sabaredzovic, Emily Deng, Madison Candela, Hunter Smith, Diana Hobbs, Jalen Scott, Johannes Levin, Chengjie Xiong, Peter Wang, Xiong Xu, Yan Li, Emily Gremminger, Yinjiao Ma, Ryan Bui, Ruijin Lu, Ralph Martins, Ana Luisa Sosa Ortiz, Alisha Daniels, Laura Courtney, Hiroshi Mori, Charlene Supnet-Bell, Jinbin Xu, John Ringman.
